# Evaluation of biotechnological processing through solid-state fermentation of oilseed cakes on extracts bioactive potential

**DOI:** 10.1007/s10529-023-03417-4

**Published:** 2023-08-11

**Authors:** Daniel Sousa, Luara Simões, Rui Oliveira, José Manuel Salgado, Maria Cambra-López, Isabel Belo, Alberto Dias

**Affiliations:** 1 Centre of Biological Engineering, University of Minho, Campus de Gualtar, 4710–057 Braga, Portugal; 2 LABBELS–Associate Laboratory, Braga, Guimarães, Portugal; 3 Institute of Animal Science Technology, Universitat Politècnica de València, Valencia, Spain; 4 Centre of Molecular and Environmental Biology, University of Minho, Braga, Portugal; 5 Biotecnia Group, Department of Chemical Engineering, University of Vigo, Campus Agua, As Lagoas S/N, 32004 Ourense, Spain

**Keywords:** Antigenotoxic, Antimicrobial, Antioxidant, Bioactive compounds, Extraction solvent, Oil cake

## Abstract

Oilseed cakes (OC) are natural sources of lignocellulosic biomass, produced every year in large amounts. In addition to their main applications as animal feed, plant or soil fertilizer, and compost, they present enormous potential for being used in biotechnological processes for the obtainment and extraction of valuable bioactive compounds. This work evaluated the effect of solid-state fermentation on the bioactive properties of extracts obtained from the bioprocessing of OC and evaluated the effect of solvents on the recovery of compounds with higher bioactive potential. A general decrease of EC_50_ values was observed for fermented extracts obtained using a mixture of water/methanol (1:1) as extraction solvent. A decrease in the minimum inhibitory concentration was observed for fermented water extracts compared to non-fermented. Additionally, growth inhibition of *Listeria monocytogenes* was observed when using aqueous methanolic fermented extracts. These extracts also exhibited a higher percentage of growth reduction against phytopathogenic fungi, and some extracts exhibited increased protection against genotoxic agents such as camptothecin and bisphenol A. It was demonstrated that bioprocessing of OC through SSF is an effective approach to obtaining valuable compounds with bioactive properties for use in the food, pharmaceutical or cosmetic industries.

## Introduction

Lignocellulosic biomass (LB) is a reliable source of material for biotechnological processing for the obtainment of bioactive compounds. LB comprises a complex matrix, in which cellulose is wrapped by the dense structure formed by hemicellulose and lignin (Yousuf et al. 2020). Cellulose is the most abundant natural biopolymer, composed of a linear chain of β–D–glucopyranose units (Coz et al. 2016). Following, hemicellulose appears as the second largest natural biopolymer with an amorphous structure, more hydrophilic than cellulose, and easier to hydrolyze (Galbe and Wallberg 2019). On the other hand, lignin is the major non-carbohydrate compound of LB, it is a heterogeneous tree-dimensional network composed of a complex structure of aromatic compounds associated with both cellulose and hemicellulose. These fractions establish non-covalent bonds and covalent cross-linkages conferring the hard structure and stability, characteristic of lignocellulosic materials, and this complex intermeshed structure makes these materials recalcitrant to enzymatic degradation (Sun et al. 2016).

A natural and abundant source of LB are the oilseed cakes (OC). The vegetable oils industry is responsible for the generation of large amounts of solid by-products called cakes, achieving a production value of 363 million tons during 2021 (OECD/FAO 2021). The OC are natural sources of LB and result from the extraction process of oil from oilseeds. OC are characterized by a high protein content, high fiber fraction and they are natural sources of minerals, vitamins, and phenolic compounds with bioactive potential properties (Švarc-Gajić et al. 2020). OC are normally used as animal feed with relative amounts of incorporation in animal feed rations, due to the presence of antinutritional compounds or high amounts of fiber that may hinder the digestive process (Greiling et al. 2018). Additionally, OC can be used as plant fertilizer, plant/soil compost, or for the obtainment of protein concentrates (Teh and Bekhit 2015). Therefore, these OC combine the perfect environment for being used as raw material in biotechnology processes. However, due to their nature, a pre-treatment process is essential to overcome their complex structure and obtain maximum profit from their exploitation.

Biotechnological processing of LB has been reported as one of the most cost-effective ways to up-cycle by-products to obtain and recover valuable bioactive compounds (protein, peptides, enzymes, pigments, phenolic compounds) with economical and industrial interest while simultaneously obtaining nutritionally up-graded products (Martínez-Espinosa 2020; Sadh et al. 2018; Sousa et al. 2022). Bioactive compounds are naturally occurring compounds, specially found in plants and food products, these compounds, present in residues matrices, can be potentially used for the prevention and/or treatment of diseases including diabetes, neurological diseases, and cardiovascular diseases (Mohd Sairazi and Sirajudeen 2020; Rangel-Huerta et al. 2015; Valencia-Mejía et al. 2019). Also, these compounds can be used for incorporation in food products contributing to the increase of the nutritional, sensorial, and technological characteristics of a certain product (Egea et al. 2018; Guimarães et al. 2019).

The use of low-environmental impact bioprocesses such as solid-state fermentation (SSF) allows the depolymerization of the lignocellulosic matrix into more simple structures. SSF is a fermentation process that mimics the conditions present in natural *habitats* and in which microorganisms grow in the absence or near absence of free water to which is applied a solid substrate used as support for the growth and development of microorganisms (Peralta et al. 2017). The use of OC as a substrate in SSF can be an effective strategy for the obtainment and recovery of valuable bioactive compounds.

Phenolic compounds are among the secondary metabolites produced by plants of greatest interest due to their bioactive properties such as antioxidant, antimicrobial, anti-inflammatory, and anti-carcinogenic (Tanase et al. 2019). The phenolic profile of LB sources such as the OC may vary depending on the oilseed variety, growing conditions, and location and can include various chemical structures such as tocopherols, flavonoids, lignans, phenolic acids, and tannins. Soybean cake (SBC), rapeseed cake (RSC), and sunflower cake (SFC) are the most produced OC at a global scale and chlorogenic acid, caffeic acid, synaptic acid, *p*-coumaric, trans-ferulic acid, hydroxybenzoic acid and isoflavones such as genistein and daidzein are the main compounds found in these OC (Nehmeh et al. 2022; Shahidi and Ambigaipalan 2015).

The objective of this work was to evaluate the effect of SSF on the bioactive properties of OC. Following previous studies, an optimized substrate composed of a mixture of OC (SFC/RSC/SBC (1:1:1)) was fermented with *Aspergillus niger* according to the work described by Sousa et al. (2022). Additionally, the effect of SSF by microbial consortiums on OC bioactive properties was studied using the combination of *Rhyzopus oryzae* and *Aspergillus ibericus*, using RSC and SFC as substrate for SSF, following the work described by Sousa et al. (2023). Additionally, the use of different extraction solvents was evaluated considering the bioactive properties of the final extracts. All the species used in this work are recognized as GRAS (generally recognized as safe) by the US Food and Drug Administration (FDA) and do not produce relevant micotoxins.

## Materials and methods

### Oilseed cakes

**Fig. 1:**
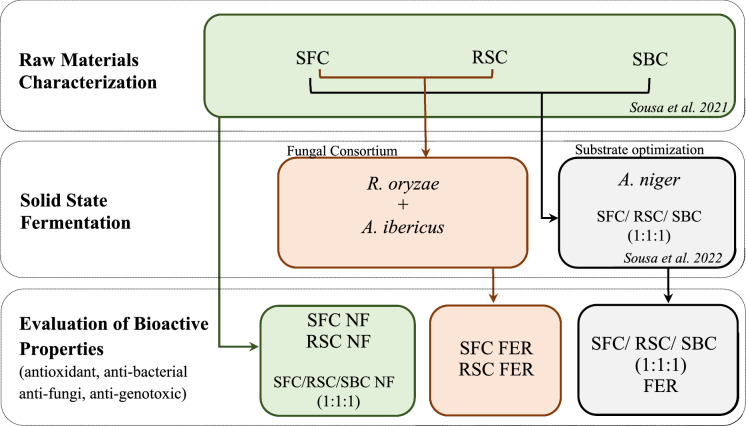
Schematic representation of extracts in study and the processes of how they were obtained. *SFC* sunflower cake, *RSC* rapeseed cake, *SBC* soybean cake, *NF* non-fermented, *FER* fermented, *A. ibericus* *Aspergillus ibericus*, *R. oryzae* *Rhyzopus oryzae*, *A. niger*, *Aspergillus niger*

Three oilseed cakes (OC) from the vegetable oils industry were used during this work: sunflower cake (SFC), rapeseed cake (RSC), and soybean cake (SBC). OC were obtained after cold-press extraction of oil and provided by industries operating in Portugal. SFC was provided by Sorgal, S. A., and RSC and SBC were provided by Iberol—*Sociedade Ibérica de Oleaginosas*, SARL. OC were dried for 24 h at 65 °C and stored in hermetic bags in the dark at 20 °C. OC were previously characterized by Sousa et al. (2021). Following previous studies, three conditions were tested during this work and the effect of solid-state fermentation (SSF) on the bioactive properties of OC extracts was evaluated by comparing the bioactivities of extracts obtained from non-fermented OC and solid-state fermented OC. The extracts used during this work and the process to obtain them are identified in Fig. 1. SFC and RSC were used to obtain extracts as well as its fermented biomass obtained by SSF with a microbial consortium composed of *Aspergillus ibericus* MUM 03.113 and *Rhyzopus oryzae* MUM 10.260. Additionally, an optimum substrate that maximizes the production of lignocellulolytic enzymes while decreasing the fiber content of biomass, composed by the conjugation of OC (SFC/RSC/SBC in a ratio of 1:1:1) was also evaluated during this work (Sousa et al. 2022). The non-fermented mixture of substrates was used for the obtainment of extracts as well as the fermented biomass obtained after SSF with *A. niger* CECT 2915 as described by Sousa et al. (2022).

### Tested extracts

A liquid/solid extraction was performed for the obtainment of liquid extracts. Extractions were performed in 1 l Erlenmeyer flasks containing 50 g of biomass. Water at 50 °C and a mixture of water/methanol (1:1) were used as extraction solvents in a ratio of 1:8 (solid: liquid). Mixtures were stirred for 1 h in an orbital shaker at 20 °C and 150 rotations per minute. The mixture was filtered with a fine-mesh net and the remaining liquid fraction was centrifuged at 2264 g for 10 min at 4 °C. Methanol present in extracts obtained using a mixture of water and methanol as solvent was recovered using a rotary evaporator at 40 °C. All extracts were freeze dried and stored in a desiccator until further use.

### In vitro antioxidant activity

For the in vitro evaluation of the antioxidant potential, the lyophilized samples were resuspended in methanol and the antioxidant potential was assessed by five different assays using concentrations varying from 6.25 to 2000 mg l^−1^. The scavenging potential of free radicals was evaluated by the DPPH method as described by Dulf et al. (2015). Extracts capacity to chelate the ferrous ion was evaluated by the iron chelating activity (ICA) and the extracts reduction potential was evaluated by the Ferric Reducing Antioxidant Power (FRAP) assay as described by Sousa et al. (2021), with some modifications. The superoxide radical scavenging potential was evaluated by the PMS-NAD non-enzymatic assay as described by Gangwar et al. (2014). These assays were performed with some modifications as described by (Sousa et al. 2021). The scavenging potential of nitric oxide (NO) was evaluated according to Sarwar et al. (2015), with some modifications. Briefly, 50 μl of sample was mixture with 50 μl of 10 mM sodium nitroprusside (SNP) in phosphate buffer saline (PBS) and incubated for 140 min at room temperature. Then, 50 μl of Griess reagent (1% sulphanilamide, 0.1% naphthylethylenediamine dichloride (NED), and 2% phosphoric acid) was added to samples and were kept in the dark for 10 min. Absorbance was measured for all assays using a spectrophotometer (SpectraMax Plus 384, Thermo-Fisher Scientific), and the results of extracts antioxidant potential were expressed as the concentration able to produce 50% of the maximal response (EC 50).

### Microdilution broth assay

Extracts were resuspended in ultra-pure sterile water, filtered through sterilized syringe filters (0.20 μm), and stored in sterile amber glass vials. Extracts antibacterial activity was tested against four pathogenic bacteria: *Bacillus subtilis* 48,886, *Escherichia coli* CECT 426, *Listeria monocytogenes* CECT 4031 T, and *Staphylococcus aureus* ATCC 25923. The minimal inhibitory concentration (MIC) was evaluated using a microdilution broth assay according to Wiegand et al. (2008). The bacterial isolates to be tested were first grown in nutrient-rich agar Mueller–Hinton (Becton–Dickinson) to obtain single colonies. Three to five isolated colonies were then transferred to Mueller–Hinton broth and incubated overnight with agitation (200 rpm) at 37 °C and *B. subtilis* at 30 °C. After incubation, suspensions were centrifuged for 3 min at 4400 g. The supernatant was discharged, and cells were washed and vortexed with a sterile saline solution NaCl 0.85% (w/w). The suspension was centrifuged for 3 min at 4400 g and this procedure was repeated one more time. Cells were resuspended in Mueller–Hinton broth and the OD of the final suspension was adjusted to 0.08–0.13 at 625 nm. After OD adjustment, bacterial suspension was used within 30 min to prevent bacterial cell growth. In a microtiter plate, 150 μl of extract was added to each well and inoculated with 150 μl of the bacterial suspension. Microtiter plates were agitated to involve the extract and bacterial suspension and kept overnight at constant shaking (200 rpm) in a temperature-controlled incubator at 37 °C or 30 °C in the case of *B. subtilis*. Additionally, in the microtiter plates, a growth control was included with 100 μl of bacterial suspension and 50 μl of Mueller–Hinton broth and a sterility control with 150 μl of Mueller–Hinton broth. For each extract were tested concentrations ranging from 0.78—25 g l^−1^. After 16–20 h of incubation and if no contamination was observed, 20 μl of resazurin reagent (R7017, Sigma-Aldrich) was added to samples (Pereira et al. 2014). The plates were then placed in an incubator at 30 or 37 °C for 2 h, all tests were performed in triplicate and the MIC was visually assessed by the color change of resazurin in each well (blue to pink in the presence of bacterial growth) (Ohikhena et al. 2017).

### Antifungal activity assay

The effect of extracts on the mycelial growth of phytopathogenic fungi (*Botrytis cinerea*, *Colletotrichum nymphaeae* 15–019, *Diplodia corticola* CAA500, *Phytophthora cinnamomi* PH107) was evaluated in Petri dishes through its incorporation in the medium (Schmitz 1930). Extracts were added to PDA medium (Biolife Italiana, S.R.L.) at approximately 50 °C at a concentration of 1500 mg l^−1^ and the final mixture was placed in 90 mm diameter sterilized Petri dishes. After agar solidification, inoculation with fungi was made with a 5 mm diameter mycelial disc of each fungus, removed from the margins of 12 days old cultures in PDA, in the center of the agar surface and incubated at 25 °C for 7 days. Water was used as negative control and added to PDA in the same amount as extracts. After 7 days of incubation, the diameter of fungi colonies was measured, and the inhibitory activity of each treatment was expressed as the percentage growth inhibition compared to the negative control using the following formula where DC = diameter of control, and DT = diameter of the fungal colony with treatment (Pandey et al. 1982):

$$growth\,inhibition \,\left(\%\right)= \frac{DC-DT}{DC}\times100$$

### Antigenotoxic potential assay

The antigenotoxic potential of extracts was evaluated by a viability assay through the spot assay according to Fauzya et al. (2019), with some modifications. The yeast culture of *Schizosaccharomyces pombe* wild type (wt) 972 h- was prepared by inoculating a loop of yeast colony in YES liquid medium (5 g/L yeast extract, 30 g/L glucose, 0.225 g L^−1^ adenine, 0.225 g L-histidine l^−1^, 0.225 g L-leucine l^−1^, 0.225 g uracil l^−1^, 0.225 g l-lysine hydrochloride l^−1^). The culture was then incubated for 24 h in a shaker at 30 °C and 200 rpm and used as a subculture. The turbidity of the subculture was then measured with a final OD_600_ of 0.4, to prepare the different conditions.

Aliquots were prepared for the different treatments: Positive control (yeast cells only in YES); Negative control (yeast cells in YES + 0.750 g methanol l^−1^); Genotoxic control (yeast cells in YES + 0.05 g camptothecin L^−1^ or 0.75 mM bisphenol A); and the treatments (yeast cells in YES + genotoxic agent + extracts). Yeast cultures were grown in the presence of 0.75 g L^−1^ of each aqueous methanolic extract (fermented or non-fermented extract of sunflower, rapeseed, or soybean cake). Cells were incubated at 30 °C for 6 h at 200 rpm, the different cell suspensions were serially diluted up to 10^–4^, and 5 µl of each dilution was then placed on top of YES agar and further incubated at 30 °C for 72 h. A preliminary evaluation of the yeast growth in the presence of each extract was made, using Petri dishes.

### Statistical analysis

Differences in extracts obtained from the same OC for evaluation of the effect of SSF were analyzed using one-way analysis of variance (ANOVA) at a significance level of 5%. All dependent variables were compared by a post hoc Tukey’s Honestly Significant Difference (HSD) test, using Statgraphics Plus 5.1 (Manusgistics, Inc., Rockville, MD). The EC50 value was obtained using GraphPad Prism software.

## Results and discussion

### Antioxidant potential of non-fermented and fermented extracts

**Fig. 2:**
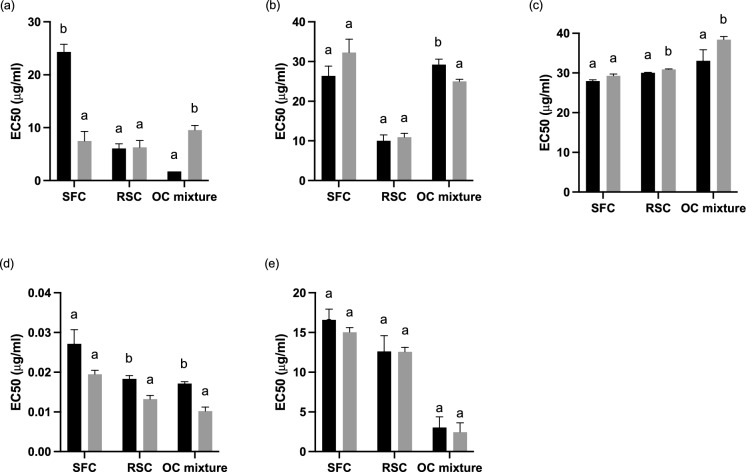
Antioxidant potential of non-fermented () and fermented aqueous extracts (). **a** DPPH radical scavenging activity, **b** iron chelation ability, **c** superoxide radical scavenging activity, **d** reducing ability **e** nitric oxide scavenging potential. SFC, sunflower cake; RSC, rapeseed cake; OC mixture, SFC/RSC/SBC (1:1:1). Results represent the average of three independent experiments and error bars represent standard deviation. Bars with equal letters for the same substrate are not statistically different (Tukey test; P < 0.05)

**Fig. 3:**
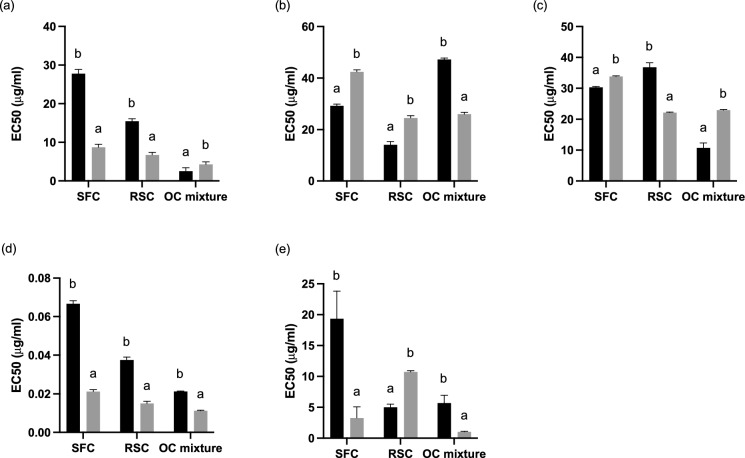
Antioxidant potential of non-fermented () and fermented aqueous methanolic extracts (). **a** DPPH radical scavenging activity, **b** iron chelation ability, **c** superoxide radical scavenging activity, **d** reducing ability **e** nitric oxide scavenging potential. SFC, sunflower cake; RSC, rapeseed cake; OC mixture, SFC/RSC/SBC (1:1:1). Results represent the average of three independent experiments and error bars represent standard deviation. Bars with equal letters for the same substrate are not statistically different (Tukey test; P < 0.05)

The effect of SSF and extraction solvent on the antioxidant potential of non-fermented and fermented extracts was evaluated. Figures 2 and 3 show the results of the effective concentrations able to produce 50% of the maximal response for the different extracts (EC 50). Considering the extracts obtained using water as extraction solvent (Fig. 2), the positive effect of SSF in the increase of antioxidant potential was clearly observed using SFC as substrate. There was a decrease of 3.25-fold of the DPPH EC 50 comparing the SFC NF with SFC FER and this decrease is translated into a higher antioxidant potential. The other antioxidant activities showed no statistically significant differences (P < 0.05) between extracts from non-fermented and fermented OC. Some variations of the antioxidant profile of RSC and the OC mixture were observed. SSF lead to an increase of the EC 50 value of superoxide scavenging potential when compared to RSC NF. On the other hand, with SSF the extracts reducing capacity increased resulting in a lower EC 50 compared to RSC NF.

Considering the mixture of OC (SFC: RSC: SBC) as the substrate for SSF, the scavenging potential of nitric oxide was the only antioxidant property that did not suffer a statistically significant variation with SSF. The NF mixture of OC exhibited a 5.46-fold higher scavenging potential of free radicals as well as a 1.16-fold higher scavenging of superoxide radicals compared to fermented extracts. On the other hand, fermented extracts exhibited a 1.17-fold higher iron chelation potential than the NF mixture of OC and a 1.7-fold higher reduction potential.

The use of organic solvents or the mixture of different solvents for the extraction of phenolic compounds with bioactive properties has been reported by several authors (Iloki-Assanga et al. 2015; Metrouh-Amir et al. 2015; Zhang et al. 2018). The use of a mixture of water/methanol (1:1) as extraction solvent resulted in an overall antioxidant profile with lower EC 50 values for fermented extracts compared to non-fermented (Fig. 3).

With SSF the scavenging of free radicals, reduction power, and scavenging of nitric oxide radical of SFC FER were significantly (P < 0.05) increased, resulting in a lower EC 50 value for each antioxidant property, compared to SFC NF extracts. The EC 50 of SFC FER was 3.18, 3.19, and 6.16-fold lower for the DPPH, FRAP, and ON when compared to the respective SFC NF EC 50 values. Contrarily, SSF led to an increase of 1.45 and 1.12-fold of the EC 50 value of iron chelation potential and scavenging potential of the superoxide radical, respectively. The antioxidant profile of RSC shows increases in the scavenging potential of free radicals and superoxide radicals and a higher reduction potential of 2.29, 1.66, and 2.53-fold, respectively. On the contrary, the chelation potential and scavenging potential of the nitric oxide radical decreased 1.75 and 2.13-fold compared to RSC NF extracts. Non-fermented extracts obtained from the mixture of OC showed 1.66 and 2.13-fold higher scavenging potential of free radicals as well as superoxide anion, respectively compared to fermented extracts. However, the last presented 1.75, 1.91, and 5.52-fold higher chelation potential, higher reduction power, and higher scavenging potential of nitric oxide, respectively. The SSF promoted variations in the antioxidant profile between non-fermented and fermented extracts that can be attributed to the deconstruction of the lignocellulosic matrix by fungi during SSF (Verduzco-Oliva and Gutierrez-Uribe 2020). The vegetable matrix is rich in phenolic compounds, in special lignin. The action of exogenous enzymes may have contributed to the release of bound phenolics and lower molecular weight compounds with higher antioxidant potential (Bhanja Dey and Kuhad 2014). Additionally, during fermentation the degree of glycosylation of phenolics changes affecting its bioactivity (Verduzco-Oliva and Gutierrez-Uribe 2020).

Overall, EC 50 values obtained using water as extraction solvent were lower than the EC 50 values obtained by conjugation of water/ methanol (1:1). A lower value of EC 50 indicates a lower extract concentration to promote 50% of the maximum response which can be translated into a higher potency of the extract compared to one with higher EC 50 values. However, it was observed statistically significant differences between extracts from NF and solid-state fermented OC for all the antioxidant assays, using a mixture of solvents. This fact indicates that the conjugation of water with methanol allowed the recovery of lower polarity species that were not recovered during the water extraction. Magwaza et al. (2016) reported that the use of conjugated solvents, more precisely de use of acidic aqueous methanol (methanol/H_2_O/HCl; 70:29.5:0.5 v/v/v), was a rapid and efficient way of recovering phenolic acids and flavone glycosides with higher yields, compared to the use of isolated solvents. Boeing et al. (2014) also reported that the use of organic-solvent water mixtures was more effective in the extraction of antioxidant compounds than their respective pure organic solvents, with acid or not. Phenolic compounds are normally responsible for the antioxidant potential of plant materials and they are mostly classified as hydrophilic antioxidants (Zhao et al. 2014). The use of methanol in conjugation with water as an extraction solvent may have allowed the extraction of medium and low molecular weight polyphenols with antioxidant potential that were not recovered during the water extraction (Dai and Mumper 2010).

### Antibacterial potential of non-fermented and fermented extracts

The bacterial inhibition potential of the extracts was evaluated against three pathogenic bacteria and *B. subtilis* through a microdilution broth assay with the incorporation of resazurin, and Table 1 depicts the results obtained. Aqueous extracts did not present any protection against three of the studied bacteria (*E. coli*, *L. monocytogenes,* and *S. aureus*), presenting an inhibitory effect only on the microbial growth of *B. subtilis*. At an effective concentration of 12.5 g l^−1^, both SFC NF and the NF mixture of substrates inhibited the microbial growth of *B. subtilis* while RSC NF extract showed higher efficiency of growth inhibition with a minimum inhibitory concentration (MIC) of 6.25 g l^−1^. Fermented aqueous extracts presented the same MIC of 3.13 g l^−1^ for *B. subitilis*. These results indicate that SSF led to a decrease in the MIC, able to inhibit the growth of the bacteria *B. subtilis*.

**Table 1:** Antibacterial potential of non-fermented (NF) and fermented (Fer) extracts of oilseed cakes using microdilution broth assay. The represented values are the minimum extract concentration (g l^−1^) able to inhibit bacterial growth. The absence of a concentration value means that the extract did not have any effect on bacterial growth within the used concentrations

		Microorganism			
Solvent	Extract	*B. subtilis*	*E. coli*	*L. monocytogenes*	*S. aureus*
Water	SFC NF	12.5	–	–	–
	SCF FER	3.125	–	–	–
	RSC NF	6.25	–	–	–
	RSC FER	3.125	–	–	–
	OC mixture NF	12.5	–	–	–
	OC mixture FER	3.125	–	–	–
Water/Methanol (1:1)	SFC NF	1.5625	–	–	–
	SCF FER	6.25	–	12.5	–
	RSC NF	6.25	–	–	–
	RSC FER	6.25	–	1.5625	–
	OC mixture NF	6.25	6.25	–	–
	OC mixture FER	6.25	3.125	12.5	–

The aqueous methanolic extracts showed growth inhibition for the bacteria *B. subtilis*, *E. coli,* and *L. monocytogenes*. The MIC observed for *B. subtilis* was 1.56 g l^−1^ for SFC NF, while the other extracts showed a MIC equal to or higher than 6.25 g l^−1^. Also, it was not observed differences between non-fermented and fermented extracts of RSC and the mixture of OC (SFC/RSC/SBC). However, SFC FER showed a MIC fourfold higher than SFC NF, indicating that there was a loss of bacterial inhibitory potential. This fact may not be necessarily related to SSF as in aqueous extracts it was observed a reduction of MIC between all NF extracts and fermented ones. The aqueous methanolic extract obtained by the conjugation of the three substrates was only able to inhibit the microbial growth of *E. coli*, and it was observed a reduction of the MIC from 6.25 g l^−1^ (non-fermented) to 3.13 g l^−1^ (fermented extracts). Non-fermented aqueous methanolic extracts were not able to have an effect on the growth inhibition of *L. monocytogenes*. Even though, fermented aqueous methanolic extracts were able to inhibit the growth of this microorganism. SFC FER and OC mixture FER showed similar values of MIC while RSC FER showed the lowest value of inhibitory concentration.

Overall, the aqueous methanolic extracts showed higher growth inhibition against the bacteria in study and, in the majority of the cases, SSF was able to increase the antibacterial effect of extracts. The use of OC as a substrate in biotechnology processes for the production of antibiotics has been previously reported. Antibiotics can be produced by different microorganisms which selectively kill or inhibit growth in low concentrations (Ancua and Sonia 2020). Sunflower, soybean, and sesame oilseed cakes were used as substrates for SSF to produce clavulanic acid and cephamycin (Gupta et al. 2018). The fungi belonging to the gender *Aspergillus* and *Rhyzopus* used in SSF for the obtainment of the extracts in the study have been previously reported due to their antimicrobial potential (Chamkhi et al. 2018; Geetanjali et al. 2018; Zhang et al. 2016). During the fermentative process, fungi are able to produce compounds with antimicrobial activity which results in the higher antimicrobial potential observed for the extracts in the study, compared to non-fermented ones (Dong et al. 2016; Kreling et al. 2020; Lourenço et al. 2018; Mohamed et al. 2016). Feitosa et al. (2020) reported the antimicrobial activity of fermented extracts against *B. subtilis* after SSF of moringa leaves flour with the fungus *A. niger* while the unfermented extracts did not exhibit the same bioactivity. The bioactive compounds produced by the studied strains showed anti-bacterial effects against both Gram-positive and Gram-negative bacteria, despite the complex structure and arrangement of the Gram-negative cell wall compared to Gram-positive bacteria. These results indicate that the studied extracts show a high antimicrobial spectrum which increases their biotechnological applications.

### Antifungal potential of non-fermented and fermented extracts

Non-fermented and fermented extracts revealed antifungal properties against the phytopathogenic fungus *Botrytis cinerea*, *Colletotrichum nymphaea*, *Diplodia corticola,* and *Phytophthora cinnamomi*. The values of the percentage of growth inhibition induced by each extract are represented in Table 2. RSC NF was the only aqueous extract showing antifungal properties against the fungus *B. cinerea* leading to a halo reduction of 11%. On the other hand, the aqueous methanolic extract obtained from RSC NF did not show antifungal properties, while the fermented (RSC FER), was able to inhibit the fungus growth by 61%, being the maximum inhibition growth observed for this fungus. The higher percentage values of growth inhibition for *C. nymphae* and *D. corticola* were observed in both RSC fermented extracts namely in the aqueous and aqueous methanolic. Also, RSC FER aqueous methanolic extract led to a higher percentage of growth inhibition of *D. corticola* and *P. cinnamomi*.

**Table 2:** Antifungal activity of non-fermented (NF) and fermented (Fer) extracts of oilseed cakes. Values represent the reduction percentage between the halo observed in Petri plates with each fungus in the presence of OC extracts against the control (fungus without extract). The absence of values indicates that the extract did not present an antifungal effect

		Microorganisms			
Extraction	Extract	*Botrytis cinerea*	*Colletotrichum nymphaea*	*Diplodia corticola*	*Phytophthora cinnamomi*
Water	SFC NF	–	17	10	–
	SCF FER	–	–	–	–
	RSC NF	11	13	10	7
	RSC FER	–	31	13	21
	OC mixture NF	–	16	14	13
	OC mixture FER	–	22	13	–
Water/Methanol (1:1)	SFC NF	–	18	16	–
	SCF FER	–	–	–	–
	RSC NF	–	21	11	–
	RSC FER	61	48	54	43
	OC mixture NF	20	11	–	–
	OC mixture FER	–	–	–	20

According to the fermentation process, the fermented extracts exhibited a higher percentage of growth inhibition compared to the respective non-fermented extracts. SSF had a positive effect in the increase of this extract's antifungal properties, being observed by a 2.4-fold higher growth inhibition percentage from the aqueous extract of RSC FER compared to the non-fermented extract for *C. nymphae*. The aqueous extract obtained from the fermented mixture of OC showed a percentage of growth inhibition 1.4-fold higher than the corresponding non-fermented extract for *C. nymphae*. Aqueous methanolic extracts showed higher fungal inhibition potential than aqueous extracts. RSC FER was able to induce fungal inhibition growth 2.3 and 4.9-fold higher than RSC NF for *C. nymphae* and *D. corticola*, respectively. The increase of antifungal properties as a consequence of SSF was also observed for the phytopathogenic fungus *P. cinnamomi* in RSC aqueous extracts where aqueous RSC FER extracts induced an inhibition growth threefold higher than RSC NF. Therefore, the effect of SSF in the increase of extracts potential against phytopathogenic fungi is clearer in RSC extracts and in extracts obtained from the mixture of OC. The non-fermented extract of SFC was the only able to confer protection against two of the fungi in the study (*C. nymphaea* and *D. corticola*).

Phytopathogenic fungi are plant invaders that may lead to plant diseases affecting crop yield and quality and are an increasing concern for the development of sustainable agriculture (Marín-Menguiano et al. 2019). The obtainment of natural extracts with potential anti-fungal activity may decrease the application of fungicides of chemical origin which are related to environmental and health problems (Leyva Salas et al. 2017). SSF has been used for the obtainment of fermented extracts with anti-fungal properties using a variety of substrates. Christ-Ribeiro et al. (2019) reported a 39.8% of inhibition growth of the fungi *Penicillium verrucosum*, after SSF of rice bran with the fungus *R. oryzae*. Denardi‐Souza et al. (2018) also reported anti-fungal properties of solid-state fermented extracts of rice bran with the fungus *R. oryzae*, against the genera *Aspergillus*, *Penicillium*, and *Fusarium*. The bioprocessing of oilseed cakes led to an increase in the anti-fungal activity of extracts increasing their potential applications namely in the agriculture area to act as plant protectors against phytopathogenic fungi. These results highlight the importance of bioprocesses for obtaining value-added products with industrial applications and the potential of OC as raw materials.

### Antigenotoxic potential of non-fermented and fermented extracts

**Fig. 4:**
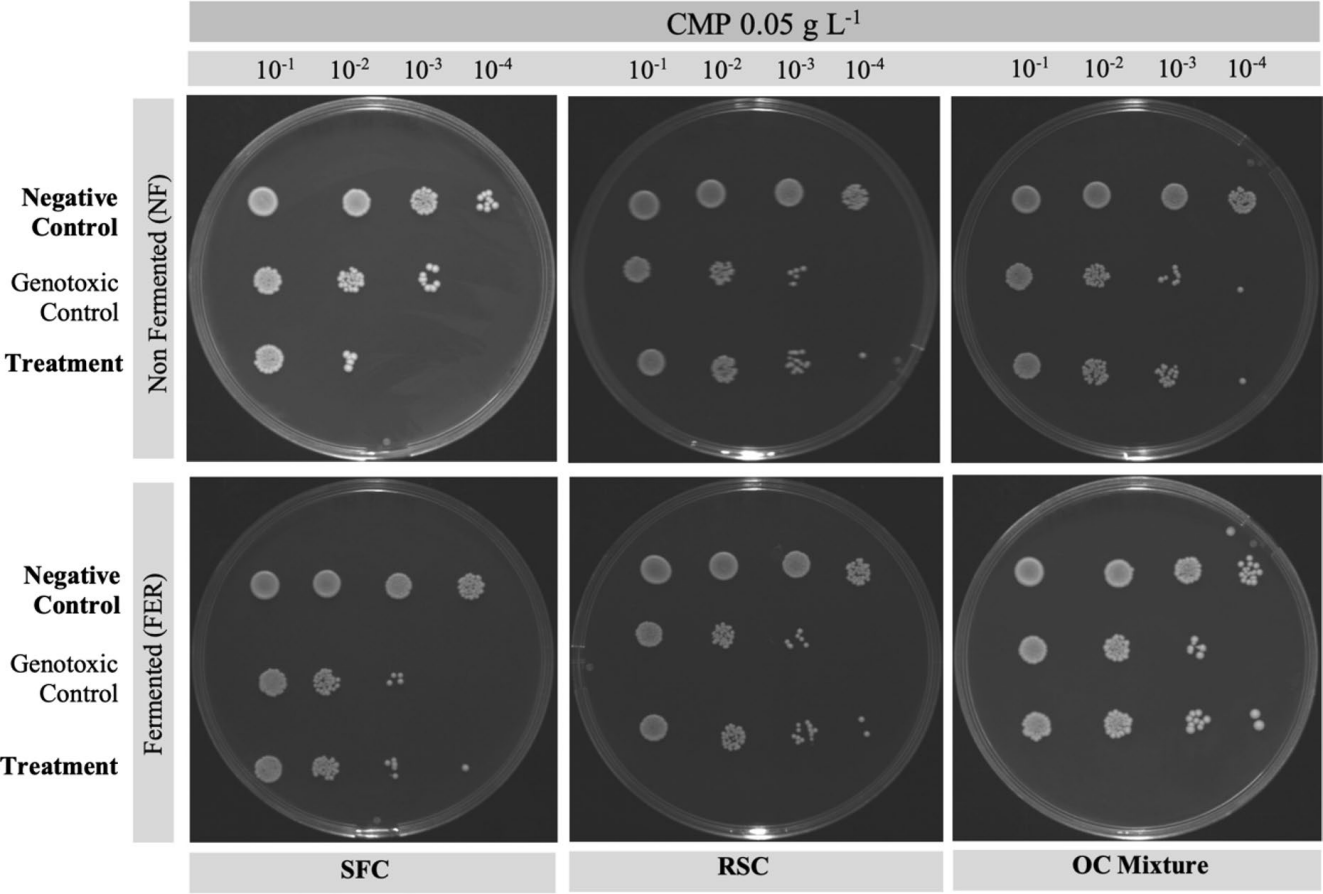
Spot assay used in viability assay performed with *Schizosaccharomyces pombe* in the presence of camptothecin and non-fermented or fermented oilseed cake extracts. Cells were grown at 30 °C for 6 h at 200 rpm. Positive control contained yeast cells grown in YES medium; Genotoxic control contained yeast cells in YES medium and 0.05 g l^−1^ of camptothecin; the Treatment contained yeast cells in YES medium, the genotoxic agent and 0.75 g l^−1^ of extract. Each figure represents the test of a different extract, and each column represents serial dilutions from 10^–1^ until 10^–4^. The photographs shown are representative of three independent experiments

**Fig. 5:**
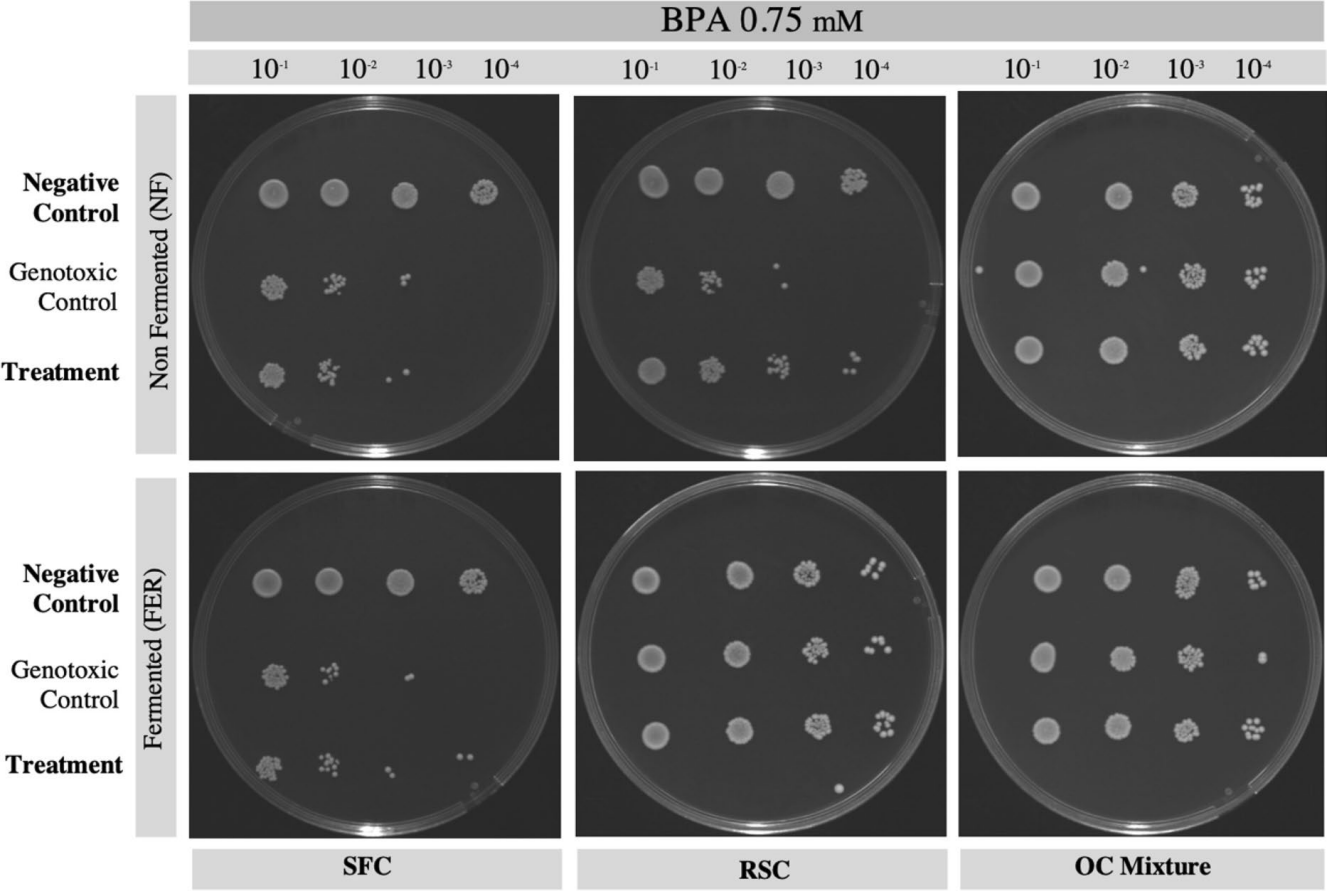
Spot assay used in viability assay performed with *Schizosaccharomyces pombe* in the presence of bisphenol A and non-fermented or fermented oilseed cake extracts. Cells were grown at 30 °C for 6 h at 200 rpm. Positive control contained yeast cells grown in YES medium; Genotoxic control contained yeast cells in YES medium and 0.75 mM of bisphenol A; the Treatment contained yeast cells in YES medium, the genotoxic agent, and 0.75 g l^−1^ of extract. Each figure represents the test of a different extract, and each column represents serial dilutions from 10^–1^ until 10^–4^. The photographs shown are representative of three independent experiments

Based on the previous results of extracts' antioxidant and antimicrobial potential, the aqueous methanolic extracts were used for the evaluation of their geno-protective potential. The effect of SSF on the geno-protective effect of extracts was evaluated by comparison of the genotoxic protection of non-fermented extracts against the genotoxic protection of fermented extracts, for the same substrate used for SSF. The results of the spot assay for the genotoxic agent camptothecin are shown in Fig. 4, and for the genotoxic agent bisphenol A (BPA) the results are shown in Fig. 5. Methanol and all the tested extracts had no effect on the growth of *S. Pombe* (not shown).

As depicted in Fig. 4, non-fermented SFC extract showed an aggravation of the genotoxic effect of camptothecin. This extract, when in the presence of camptothecin, intensified the damage caused by this genotoxic agent. For the other tested extracts, the protective effect on the yeast cells was slightly noticed when in the presence of non-fermented RSC, fermented RSC, or fermented OC Mixture since some damage mitigation effect was observed in these treatments.

Considering the use of BPA as a genotoxic agent (Fig. 5), only in the presence of non-fermented RSC extract, an effect of damage mitigation was observed. In this case, it is possible to observe a higher yeast growth when in the presence of non-fermented RSC extract and the genotoxic agent BPA, compared to the genotoxic control.

Exposure to genotoxic agents may result in DNA or chromosomal damage with possible serious effects in human health such as cancer. Therefore, the search for alternative compounds with biological activity capable of mitigating the harmful effects of external DNA-damaging agents on our health, such as camptothecin and BPA, should be considered. The adverse effect caused by camptothecin affects topoisomerase I, allowing the cleavage of DNA but inhibiting the subsequent ligation resulting in DNA double-strand breaks (Attenello et al. 2012). This agent is a plant alkaloid, widely used as an anti-tumor drug in chemotherapy (Pund and Joshi 2017). Regarding BPA, this organic compound is used for the production of polycarbonate plastic and epoxy resins which are part of the packaging materials for food and beverages and can migrate to foods and the environment (Špačková et al. 2020). It is believed that BPA has endocrine disruptive effects on humans by interacting with biological receptors such as estrogen, androgen, and thyroid hormone receptors, which results in health hazards effects on the reproductive and nervous systems, metabolic function, immune function, and interferes with growth and development of offspring (EFSA Panel on Food Contact Materials Flavourings and Processing Aids (CEF) 2015; Wang et al. 2022). Besides, several authors have reported the genotoxicity of BPA namely in the impairment of the double-strand break repair mechanism and the toxicity of this agent on the yeast *S. pombe* (Aghajanpour-Mir et al. 2016; Pfeifer et al. 2015; Špačková et al. 2020). Initiatives to reduce exposure to these agents are essential to mitigate their harmful effects on the human body. However, the use of biotechnological processing for the obtainment of extracts with a geno-protective effect is additional protection. In this innovative study, some of the extracts exhibited geno-protection, being able to mitigate the effects of camptothecin and BPA widening the application of these extracts to the pharmaceutical industry.

## Conclusions

Oilseed cakes bioprocessing by SSF allowed the obtention of extracts with enhanced bioactive potential. The microbiological disruption of OC lignocellulosic matrix contributed to higher bioactive properties. The use of different extraction solvents for the obtainment of solid-state fermented OC extracts allowed to conclude that the aqueous methanolic solvent mixture recovered compounds with lower polarity that contributed to the increase of the overall protection against certain agents including microbial, genotoxic, and oxidants. This is a step further for the establishment of bioprocesses, namely SSF, as a reliable alternative for the obtainment of compounds with increased bioactive properties contributing to the up-grading of by-products and wastes within the concept of the circular economy. Deeper studies are needed to test the efficiency of extracts conferring protection of cell lines using in vitro trials against harmful agents. Nevertheless, it was demonstrated the potential of fermented extracts for industrial applications in the areas of food, cosmetics, agriculture, and pharmaceutical.

## References

[CR1] Aghajanpour-Mir SM, Zabihi E, Akhavan-Niaki H, Keyhani E, Bagherizadeh I, Biglari S, Behjati F (2016). The genotoxic and cytotoxic effects of bisphenol-A (BPA) in MCF-7 cell line and amniocytes. Int J Mol Cell Med.

[CR2] Ancuța P, Sonia A (2020). Oil Press-cakes and meals valorization through circular economy approaches: a review. Appl Sci.

[CR3] Attenello F, Raza SM, Dimeco F, Olivi A, Aminoff MJ, Boller F, Swaab N (2012). Chemotherapy for brain tumors with polymer drug delivery. Neuro-oncology.

[CR4] Bhanja Dey T, Kuhad RC (2014). Upgrading the antioxidant potential of cereals by their fungal fermentation under solid-state cultivation conditions. Lett Appl Microbiol.

[CR5] Boeing JS, Barizão ÉO, Montanher PF, de Cinque Almeida V, Visentainer JV (2014). Evaluation of solvent effect on the extraction of phenolic compounds and antioxidant capacities from the berries: application of principal component analysis. Chem Cent J.

[CR6] Chamkhi I, Sbabou L, Aurag J (2018). Endophytic fungi isolated from *Crocus sativus* L. (saffron) as a source of bioactive secondary metabolites. Pharm J.

[CR7] Christ-Ribeiro A, Graça CS, Kupski L, Badiale-Furlong E, de Souza-Soares LA (2019). Cytotoxicity, antifungal and anti mycotoxins effects of phenolic compounds from fermented rice bran and Spirulina sp.. Process Biochem.

[CR8] Coz A, Llano T, Cifrián E, Viguri J, Maican E, Sixta H (2016). Physico-chemical alternatives in lignocellulosic materials in relation to the kind of component for fermenting purposes. Materials.

[CR9] Dai J, Mumper RJ (2010). Plant phenolics: extraction, analysis and their antioxidant and anticancer properties. Molecules.

[CR10] Denardi-Souza T, Luz C, Mañes J, Badiale-Furlong E, Meca G (2018). Antifungal effect of phenolic extract of fermented rice bran with Rhizopus oryzae and its potential use in loaf bread shelf life extension. J Sci Food Agric.

[CR11] Dong J-W, Cai L, Li X-J, Duan R-T, Shu Y, Chen F-Y, Wang J-P, Zhou H, Ding Z-T (2016). Production of a new tetracyclic triterpene sulfate metabolite sambacide by solid-state cultivated Fusarium sambucinum B10.2 using potato as substrate. Biores Technol.

[CR12] Dulf FV, Vodnar DC, Dulf E-H, Toşa MI (2015). Total phenolic contents, antioxidant activities, and lipid fractions from berry pomaces obtained by solid-state fermentation of two *Sambucus species* with *Aspergillus niger*. J Agric Food Chem.

[CR13] EFSA Panel on Food Contact Materials Flavourings and Processing Aids (CEF) (2015). Scientific opinion on the risks to public health related to the presence of bisphenol A (BPA) in foodstuffs. EFSA J.

[CR14] Egea MB, Bolanho BC, Lemes AC, Bragatto MM, Silva MR, de Carvalho JCM, Danesi EDG (2018). Low cost cassava, peach palm and soy by-products for the nutritional enrichment of cookies: physical, chemical and sensorial characteristics. Int Food Res J.

[CR15] Fauzya AF, Astuti RI, Mubarik NR (2019). Effect of ethanol-derived clove leaf extract on the oxidative stress response in yeast *Schizosaccharomyces pombe*. Int J Microbiol.

[CR16] Feitosa PRB, Santos TRJ, Gualberto NC, Narain N, de Aquino Santana LCL (2020). Solid-state fermentation with *Aspergillus niger* for the bio-enrichment of bioactive compounds in *Moringa oleifera* (moringa) leaves. Biocatal Agric Biotechnol.

[CR17] Galbe M, Wallberg O (2019). Pretreatment for biorefineries: a review of common methods for efficient utilisation of lignocellulosic materials. Biotechnol Biofuels.

[CR18] Gangwar M, Gautam MK, Sharma AK, Tripathi YB, Goel RK, Nath G (2014). Antioxidant capacity and radical scavenging effect of polyphenol rich Mallotus philippenensis fruit extract on human erythrocytes: an in vitro study. Sci World J.

[CR19] Geetanjali G, Jain P, Pundir, R. K. (2018). Evaluation of antimicrobial potential of aspergillus ibericus isolated from rhizospheric soil of ficus religiosa. International Journal of Pharmacy and Biological Sciences.

[CR20] Greiling AM, Reiter R, Rodehutscord M (2018). Utilization of unprocessed and fibre-reduced oilseed cakes of rapeseed and sunflower seed in rainbow trout (*Oncorhynchus mykiss* W.) nutrition—Evaluation of apparent digestibility and growth performance. Aquac Nutr.

[CR21] Guimarães RM, Pimentel TC, de Rezende TAM, de Silva JS, Falcão HG, Ida EI, Egea MB (2019). Gluten-free bread: effect of soy and corn co-products on the quality parameters. Eur Food Res Technol.

[CR22] Gupta, A., Sharma, R., Sharma, S., & Singh, B. (2018). Oilseed as potential functional food Ingredient. In *Trends & Prospects in Food Technology, Processing and Preservation, 1st ed.; Prodyut Kumar, P., Mahawar, MK, Abobatta, W., Panja, P., Eds* (pp. 25–58).

[CR23] Iloki-Assanga SB, Lewis-Luján LM, Lara-Espinoza CL, Gil-Salido AA, Fernandez-Angulo D, Rubio-Pino JL, Haines DD (2015). Solvent effects on phytochemical constituent profiles and antioxidant activities, using four different extraction formulations for analysis of *Bucida buceras* L. and *Phoradendron californicum*. BMC Res Notes.

[CR24] Kreling NE, Simon V, Fagundes VD, Thomé A, Colla LM (2020). Simultaneous production of lipases and biosurfactants in solid-state fermentation and use in bioremediation. J Environ Eng.

[CR25] Leyva Salas M, Mounier J, Valence F, Coton M, Thierry A, Coton E (2017). Antifungal microbial agents for food biopreservation—A review. Microorganisms.

[CR26] Lourenço LA, Alberton Magina MD, Tavares LBB, Ulson G, de Souza SMA, García Román M, Altmajer Vaz D (2018). Biosurfactant production by Trametes versicolor grown on two-phase olive mill waste in solid-state fermentation. Environ Technol.

[CR27] Magwaza LS, Opara UL, Cronje PJR, Landahl S, Ortiz JO, Terry LA (2016). Rapid methods for extracting and quantifying phenolic compounds in citrus rinds. Food Sci Nutr.

[CR28] Marín-Menguiano M, Moreno-Sánchez I, Barrales RR, Fernández-Álvarez A, Ibeas JI (2019). N-glycosylation of the protein disulfide isomerase Pdi1 ensures full Ustilago maydis virulence. PLoS Pathog.

[CR29] Martínez-Espinosa RM (2020). Introductory Chapter: a brief overview on fermentation and challenges for the next future. New Adv Ferment Process.

[CR30] Metrouh-Amir H, Duarte CMM, Maiza F (2015). Solvent effect on total phenolic contents, antioxidant, and antibacterial activities of Matricaria pubescens. Ind Crops Prod.

[CR31] Mohamed SA, Saleh RM, Kabli SA, Al-Garni SM (2016). Influence of solid state fermentation by Trichoderma spp on solubility, phenolic content, antioxidant, and antimicrobial activities of commercial turmeric. Biosci Biotechnol Biochem.

[CR32] Mohd Sairazi NS, Sirajudeen KNS (2020). Natural products and their bioactive compounds: neuroprotective potentials against neurodegenerative diseases. Evidence-Based Complementary Altern Med.

[CR33] Nehmeh M, Rodriguez-Donis I, Cavaco-Soares A, Evon P, Gerbaud V, Thiebaud-Roux S (2022). Bio-refinery of oilseeds: oil extraction, secondary metabolites separation towards protein meal valorisation—a review. Processes.

[CR34] OECD/FAO (2021). OECD-FAO agricultural outlook 2021–2030. OECD.

[CR35] Ohikhena FU, Wintola OA, Afolayan AJ (2017). Evaluation of the antibacterial and antifungal properties of *Phragmanthera capitata* (Sprengel) Balle (Loranthaceae), a mistletoe growing on rubber tree, using the dilution techniques. Sci World J.

[CR36] Peralta RM, da Silva BP, Gomes Côrrea RC, Kato CG, Vicente Seixas FA, Bracht A, Brahmachari GBT-B of ME (2017). Enzymes from Basidiomycetes—Peculiar and Efficient Tools for Biotechnology. Biotechnology of Microbial Enzymes.

[CR37] Pereira V, Dias C, Vasconcelos MC, Rosa E, Saavedra MJ (2014). Antibacterial activity and synergistic effects between Eucalyptus globulus leaf residues (essential oils and extracts) and antibiotics against several isolates of respiratory tract infections (Pseudomonas aeruginosa). Ind Crops Prod.

[CR38] Pfeifer D, Chung YM, Hu MCT (2015). Effects of low-dose bisphenol A on DNA damage and proliferation of breast cells: the role of c-Myc. Environ Health Perspect.

[CR39] Pund S, Joshi A (2017). Nanoarchitectures for neglected tropical protozoal diseases: challenges and state of the art. Nano Microscale Drug Deliv Syst.

[CR40] Rangel-Huerta OD, Pastor-Villaescusa B, Aguilera CM, Gil A (2015). A systematic review of the efficacy of bioactive compounds in cardiovascular disease: phenolic compounds. Nutrients.

[CR41] Sadh PK, Kumar S, Chawla P, Duhan JS (2018). Fermentation: a boon for production of bioactive compounds by processing of food industries wastes (by-products). Molecules.

[CR42] Sarwar R, Farooq U, Khan A, Naz S, Khan S, Khan A, Rauf A, Bahadar H, Uddin R (2015). Evaluation of antioxidant, free radical scavenging, and antimicrobial activity of Quercus incana Roxb. Front Pharmacol.

[CR43] Schmitz H (1930). Poisoned food technique. Ind Eng Chem-Anal Edit.

[CR44] Shahidi F, Ambigaipalan P (2015). Phenolics and polyphenolics in foods, beverages and spices: antioxidant activity and health effects–A review. Journal of Functional Foods.

[CR45] Sousa D, Salgado JM, Cambra-López M, Dias ACP, Belo I (2021). Degradation of lignocellulosic matrix of oilseed cakes by solid state fermentation: fungi screening for enzymes production and antioxidants release. J Sci Food Agric.

[CR46] Sousa D, Salgado JM, Cambra-López M, Dias A, Belo I (2022). Biotechnological valorization of oilseed cakes: substrate optimization by simplex centroid mixture design and scale-up to tray bioreactor. Biofuels, Bioprod Biorefin.

[CR47] Sousa D, Salgado JM, Cambra-López M, Dias A, Belo I (2023). Bioprocessing of oilseed cakes by fungi consortia: impact of enzymes produced on antioxidants release. J Biotechnol.

[CR48] Špačková J, Oliveira D, Puškár M, Ďurovcová I, Gaplovská-Kyselá K, Oliveira R, Ševčovičová A (2020). Endocrine-independent cytotoxicity of bisphenol A is mediated by increased levels of reactive oxygen species and affects cell cycle progression. J Agric Food Chem.

[CR49] Sun S, Sun S, Cao X, Sun R (2016). The role of pretreatment in improving the enzymatic hydrolysis of lignocellulosic materials. Biores Technol.

[CR50] Švarc-Gajić J, Morais S, Delerue-Matos C, Vieira EF, Spigno G (2020). Valorization potential of oilseed cakes by subcritical water extraction. Appl Sci.

[CR51] Tanase C, Coșarcă S, Muntean D-L (2019). A critical review of phenolic compounds extracted from the bark of woody vascular plants and their potential biological activity. Molecules.

[CR52] Teh S-S, Bekhit AE-DA (2015). Utilization of oilseed cakes for human nutrition and health benefits. Agricultural biomass based potential materials.

[CR53] Valencia-Mejía E, Batista KA, Fernández JJA, Fernandes KF (2019). Antihyperglycemic and hypoglycemic activity of naturally occurring peptides and protein hydrolysates from easy-to-cook and hard-to-cook beans (*Phaseolus vulgaris* L.). Food Res Int.

[CR54] Verduzco-Oliva R, Gutierrez-Uribe JA (2020). Beyond enzyme production: Solid state fermentation (SSF) as an alternative approach to produce antioxidant polysaccharides. Sustainability.

[CR55] Wang X, Nag R, Brunton NP, Siddique MAB, Harrison SM, Monahan FJ, Cummins E (2022). Human health risk assessment of bisphenol A (BPA) through meat products. Environ Res.

[CR56] Wiegand I, Hilpert K, Hancock REW (2008). Agar and broth dilution methods to determine the minimal inhibitory concentration (MIC) of antimicrobial substances. Nat Protoc.

[CR57] Yousuf A, Pirozzi D, Sannino F (2020). Fundamentals of lignocellulosic biomass. Lignocellulosic biomass to liquid biofuels.

[CR58] Zhang H, Tang Y, Ruan C, Bai X (2016). Bioactive secondary metabolites from the endophytic Aspergillus Genus. Records Nat Prod.

[CR59] Zhang Q-W, Lin L-G, Ye W-C (2018). Techniques for extraction and isolation of natural products: a comprehensive review. Chinese Med.

[CR60] Zhao H, Zhang H, Yang S (2014). Phenolic compounds and its antioxidant activities in ethanolic extracts from seven cultivars of Chinese jujube. Food Sci Human Wellness.

